# Beyond Cultures: The Evolving Role of Molecular Diagnostics, Synovial Biomarkers and Artificial Intelligence in the Diagnosis of Prosthetic Joint Infections

**DOI:** 10.3390/jcm14196886

**Published:** 2025-09-29

**Authors:** Martina Maritati, Giuseppe De Rito, Gustavo Alberto Zanoli, Yu Ning, Matteo Guarino, Roberto De Giorgio, Carlo Contini, Andrej Trampuz

**Affiliations:** 1 Department of Translational Medicine, University of Ferrara, 44121 Ferrara, Italy; grnmtt@unife.it (M.G.); dgrrrt@unife.it (R.D.G.); 2 Orthopedic Ward, Casa di Cura Santa Maria Maddalena, 45030 Occhiobello, Italy; derito1974@libero.it (G.D.R.); zanolig@me.com (G.A.Z.); 3 Center for Musculoskeletal Surgery (CMSC), Charité–Universitätsmedizin Berlin, Humboldt-Universität zu Berlin, and Berlin Institute of Health, 13353 Berlin, Germany; yu.ning@charite.de; 4 Department of Medical Sciences, University of Ferrara, 44121 Ferrara, Italy; cnc@unife.it; 5 School of Medicine, Faculty of Health, Queensland University of Technology (QUT), Brisbane, QLD 4000, Australia; andrej.trampuz@qut.edu.au

**Keywords:** periprosthetic joint infection, non-culture diagnostics, molecular techniques, synovial biomarkers, pathogen-derived biomarkers, artificial intelligence, multiplex immunoassay

## Abstract

Periprosthetic joint infection (PJI) remains a major complication in orthopedic surgery, with accurate and timely diagnosis being essential for optimal patient management. Traditional culture-based diagnostics are often limited by suboptimal sensitivity, especially in biofilm-associated and low-virulence infections. In recent years, non-culture-based methodologies have gained prominence. Molecular techniques, such as polymerase chain reaction (PCR) and next-generation sequencing (NGS), offer enhanced detection of microbial DNA, even in culture-negative cases, and enable precise pathogen identification. In parallel, extensive research has focused on biomarkers, including systemic (e.g., C-reactive protein, fibrinogen, D-dimer), synovial (e.g., alpha-defensin, calprotectin, interleukins), and pathogen-derived markers (e.g., D-lactate), the latter reflecting metabolic products secreted by microorganisms during infection. The development of multiplex platforms now allows for the simultaneous measurement of multiple synovial biomarkers, improving diagnostic accuracy and turnaround time. Furthermore, the integration of artificial intelligence (AI) and machine learning algorithms into diagnostic workflows has opened new avenues for combining clinical, molecular, and biochemical data. These models can generate probability scores for PJI diagnosis with high accuracy, supporting clinical decision-making. While these technologies are still being validated for routine use, their convergence marks a significant step toward precision diagnostics in PJI, potentially improving early detection, reducing diagnostic uncertainty, and guiding targeted therapy.

## 1. Introduction

Peri-prosthetic infection (PJI) is a devastating complication of joint replacement surgery, with serious implications for patient health in terms of morbidity and mortality, and high costs to the healthcare system [1].

PJI is becoming more common worldwide as the number of joint arthroplasties performed rises over time [2], with an estimated incidence of 2% in primary [3,4,5] and 2–15% in revision arthroplasties [6].

Diagnosis of PJI remains a challenge. To date, no test with 100% diagnostic accuracy has been identified. As a result, clinicians often use a combination of intra-operative, laboratory and clinical findings. Despite this, PJI is often difficult to diagnose and might be overlooked without a high index of suspicion [7]. Several diagnostic criteria for PJI have been proposed by different societies [8,9], but none has been universally accepted as the “gold standard”.

Recently, the European Bone and Joint Infection Society (EBJIS) definition has been shown to be more sensitive than previously published criteria without compromising specificity [10]. Distinguishing PJI from aseptic failure is critical, as these two conditions require different treatments. In some cases, however, it is difficult to make a definitive diagnosis, and, consequently, choose the most appropriate treatment. This is particularly true for PJI caused by chronic low-grade inflammation, which represent the most challenging cases from a diagnostic perspective [11,12].

### 1.1. Microbiological Culture: Strengths and Limitations

Microbiological culture continues to represent the cornerstone of PJI diagnostics, enabling pathogen identification and antimicrobial susceptibility testing [13]. The most commonly analyzed samples include: synovial fluid, collected via joint aspiration, peri-prosthetic tissue, obtained intraoperatively during revision surgery, and prosthetic surfaces, often processed using techniques such as sonication to disrupt and recover as biofilm attached, sessile micro-organisms [9,14,15].

One of the major advantages of culture is that it enables definitive pathogen identification, allowing clinicians to tailor antimicrobial therapy based on the causative pathogen. In addition, once the microorganism is isolated, antimicrobial susceptibility testing can be performed, offering valuable insights into resistance patterns and guiding the selection of the most effective antibiotics. Another key strength of culture is its widespread availability; it is a standard procedure in microbiology laboratories across the world and can be performed without the need for highly specialized equipment [16].

However, culture is not without limitations, and these must be carefully considered in the diagnostic process. One significant drawback is the possibility of false-negative results, especially in patients who have received prior antibiotic therapy, or in cases where the bacterial load is low or organisms are embedded in biofilms [17]. Such scenarios can compromise the sensitivity of the method, leading to missed diagnoses. Furthermore, culture is often a time-consuming process, with some pathogens requiring several days to grow, thus delaying initiation of targeted therapy. Chronic and low-grade infections, which are commonly caused by slow-growing or fastidious organisms, may be particularly challenging to detect using conventional culture techniques [18]. Additionally, there is a risk of contamination, which can result in false-positive results and complicate clinical interpretation [19].

Although microbiological culture remains the gold standard for PJI, with a reported sensitivity ranging from 65% to 95% and a pivotal role in guiding antimicrobial therapy, it is imperative to acknowledge its inherent limitations.

**Figure 1:**
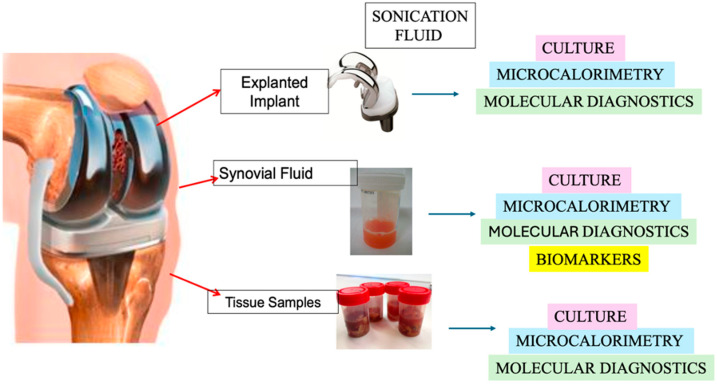
Schematic representation of the main sample types used for the diagnosis of PJI. Explanted implants are processed through sonication to obtain sonication fluid, which can be analyzed by culture, microcalorimetry, and molecular methods. Synovial fluid allows for culture, microcalorimetry, molecular diagnostics, and biomarker testing. Periprosthetic tissue samples are investigated by culture, microcalorimetry, and molecular assays.

As summarized in Figure 1, these different sample types—synovial fluid, periprosthetic tissue, and sonication fluid—can be analyzed not only by culture but also through emerging non-culture-based methodologies, which are increasingly being investigated to overcome these diagnostic challenges.

### 1.2. Emerging Diagnostic Modalities

To address these challenges, several novel diagnostic approaches have emerged.

Microcalorimetry has attracted attention as a rapid culture-independent tool. By measuring heat flow generated by microbial metabolism, it enables early detection of bacterial growth, often within hours, and may prove particularly useful for slow-growing or fastidious organisms frequently implicated in PJI [20]. Although still limited to specialized centers, microcalorimetry could complement traditional and molecular diagnostics by providing both rapid and phenotypic evidence of infection.

Molecular techniques such as quantitative polymerase chain reaction (qPCR) and multiplex PCR assays allow the detection of bacterial DNA directly from synovial fluid or periprosthetic tissue, improving sensitivity in culture-negative cases. More advanced next-generation sequencing (NGS) platforms can provide broad-range pathogen identification and even resistance gene profiling, offering a comprehensive view of the microbial landscape in PJI [21,22]. Despite their promise, these methods face challenges including cost, turnaround time, and difficulties in distinguishing contamination from true infection.

In parallel, significant progress has been made in biomarker discovery. While serum C-reactive protein (CRP) and erythrocyte sedimentation rate (ESR) remain widely used, their specificity is limited. Synovial biomarkers—including leukocyte esterase, α-defensin, calprotectin—have demonstrated higher diagnostic accuracy and are now considered valuable adjuncts in both pre- and intraoperative settings. Recent meta-analyses confirm their utility, yet issues of cost, accessibility, and standardization remain [23].

### 1.3. The Emerging Role of Artificial Intelligence

More recently, artificial intelligence (AI) has emerged as a transformative tool in the field of PJI, offering opportunities to overcome long-standing diagnostic and therapeutic challenges. Conventional approaches to PJI diagnosis are often limited by the lack of a single gold standard and by the heterogeneous performance of microbiological, molecular, and imaging modalities. AI-driven methods, including machine learning and deep learning algorithms, have shown the capacity to integrate complex datasets—ranging from clinical, laboratory, and radiographic information to molecular and histopathological profiles—to improve diagnostic precision and support clinical decision-making. Beyond diagnostics, AI applications are increasingly being investigated for their potential to stratify patient risk, predict outcomes, and optimize individualized treatment strategies. Collectively, these developments highlight the potential of AI to complement traditional methodologies and contribute to a more accurate, timely, and personalized management of PJI [24].

In this manuscript, we review the current evidence on microcalorimetry, molecular diagnostics—including NGS—synovial biomarkers, and highlight emerging AI-based approaches. We propose an integrated framework that connects these complementary modalities to improve diagnostic precision, shorten time to diagnosis, and ultimately optimize outcomes in patients with PJI.

## 2. Non-Culture Diagnostics Methods

### 2.1. Microcalorimetry

Isothermal microcalorimetry (IMC) is emerging as a promising diagnostic tool for PJI, offering a rapid and sensitive alternative to conventional culture methods. By measuring the heat produced by microbial metabolism, IMC enables real-time detection of bacterial growth, significantly reducing the time to diagnosis [20]. In a study involving 152 patients undergoing revision hip or knee arthroplasty, IMC demonstrated a sensitivity of 83% and specificity of 100%, with a median time to detection of 10 h, compared to 51 h for traditional cultures. Notably, in patients receiving chronic antibiotic therapy, IMC maintained high diagnostic accuracy, highlighting its utility in challenging clinical scenarios [25].

Furthermore, combining IMC with sonication of explanted prosthetic material has yielded sensitivity and specificity rates of 100% and 97%, respectively, with a mean detection time of approximately 11 h. These findings suggest that IMC can enhance the speed and accuracy of PJI diagnosis, potentially leading to improved patient outcomes through timely and targeted therapeutic interventions [26].

Despite its promising diagnostic performance, IMC presents several limitations that currently hinder its widespread clinical adoption in the diagnosis of PJI. First, IMC is not yet widely available in routine microbiology laboratories due to the specialized equipment required and associated costs, limiting its accessibility. Second, while IMC can detect microbial heat production rapidly, it does not provide information on pathogen identification or antimicrobial susceptibility, necessitating complementary conventional culture or molecular techniques [20,27].

Moreover, the sensitivity of IMC may be affected by the bacterial load and metabolic activity; low-grade infections or slow-growing organisms might result in delayed or false-negative results. Additionally, distinguishing between contaminant heat signals and true infection, especially in polymicrobial or biofilm-associated infections, remains a challenge requiring expert interpretation [25]. Finally, although IMC accelerates detection, it does not eliminate the need for sterile sample handling and optimized pre-analytical steps such as sonication, which may impact the reliability and reproducibility of results. These limitations underscore the need for further standardization, integration with other diagnostic modalities, and clinical validation in larger, multicentric cohorts.

Recent developments in diagnostic microbiology have demonstrated the potential of IMC with machine learning (ML) and deep learning techniques to enhance the diagnosis of PJI. The study by Lozano-García et al. represents a pivotal advance in the field, as it demonstrates for the first time the feasibility of using IMC data alone for both detection and identification of pathogens in synovial fluid. A total of 413 IMC heat flow curves—obtained from 239 PJI and 174 aseptic synovial fluid samples—were analyzed using multiple classifiers, including XGBoost, SVM, random forest, multi-layer perceptrons, and convolutional neural networks (CNNs) via transfer learning. The binary XGBoost classifier achieved a 100% accuracy for PJI detection, while the best multiclass XGBoost model and combined CNN classifier reached pathogen identification accuracies of 90.3% and 91.5%, respectively. These results affirm that specific pathogens exhibit distinct thermogenic signatures detectable through IMC, and that artificial intelligence (AI) models can effectively classify them, offering a rapid, label-free diagnostic alternative to conventional culture. This approach addresses a major limitation of IMC—its lack of species-level resolution—thus enhancing its clinical utility in guiding early and targeted antimicrobial therapy [28].

### 2.2. Molecular Methods

Molecular diagnostic techniques have emerged as valuable tools in detecting PJI, particularly in cases where traditional cultures yield negative results. These methods, including polymerase chain reaction (PCR) and next-generation sequencing (NGS), offer rapid and sensitive detection of pathogens directly from clinical specimens, potentially improving diagnostic accuracy and patient outcomes.

#### 2.2.1. Polymerase Chain Reaction (PCR)

PCR-based assays amplify specific DNA sequences of pathogens, enabling their detection even in low abundance. Two recent meta-analyses have reported pooled sensitivities and specificities for PCR in PJI diagnosis ranging from 73.3% to 86% and 91% to 95.5%, respectively [29,30].

Nonetheless, the interpretation of these results is constrained by substantial statistical heterogeneity, encompassing variations in study design, specimen type, implementation of sonication procedures, PCR protocols, and reference standards. Such heterogeneity may have contributed to an overestimation of the actual diagnostic accuracy of the method.

**Figure 2:**
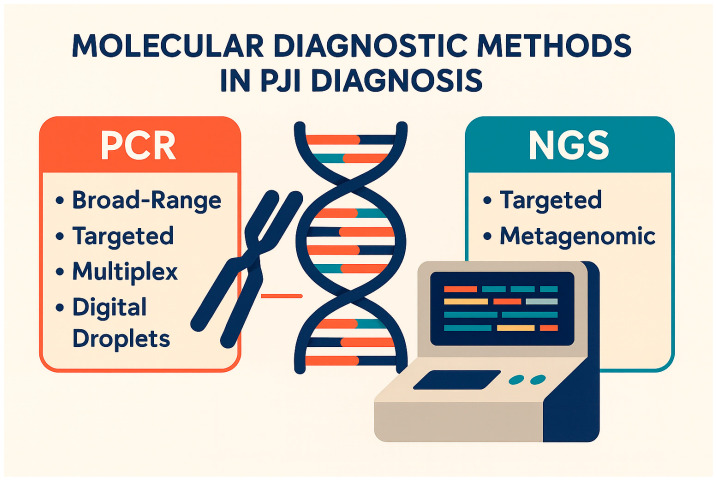
Molecular diagnostic methods in PJI.

Several PCR techniques have been employed in the diagnostic evaluation of PJI (Figure 2).

Universal or broad-range PCR (BR-PCR) is a molecular approach that employs primers targeting conserved regions of bacterial or fungal genomes, allowing for the amplification of DNA from all potential pathogens. This process is followed by sequencing to achieve species-level identification [31]. BR-PCR has demonstrated a sensitivity of 82.2% and a specificity of approximately 94% in the diagnosis of PJI. Nevertheless, it shows significant limitations in the detection of fungal and polymicrobial infections [31]. While its sensitivity is lower compared to targeted or multiplex PCR techniques, BR-PCR remains valuable for its ability to identify pathogens not previously known or recognized as causative agents of infection [32]. On the contrary, targeted PCR assays, particularly multiplex PCR (M-PCR) platforms, have demonstrated higher diagnostic performance.

M-PCR enables the simultaneous detection of multiple pathogens by amplifying various target sequences using multiple primer sets within a single reaction. In addition, it allows for the identification of specific genes encoding resistance mechanisms [33] or virulence factors (e.g., toxin production) [34,35].

M-PCR applied to synovial fluid has shown a sensitivity of approximately 84% and a specificity of 89%. Similar accuracy is maintained in sonication fluid (sensitivity ~81%, specificity ~96%) [36].

A recent study, showed an overall diagnostic performance of synovial fluid M-PCR overlapping that of conventional cultures, but enhanced sensitivity in identifying low-virulence organisms, including *Cutibacterium* species and coagulase-negative *Staphylococcus* [37].

Despite this finding, one of the most recent commercially available multiplex panels designed for the diagnosis of PJI does not include *Cutibacterium* spp. and *S. epidermidis* among its 39 targeted pathogens. As a result, it serves as a valuable tool for diagnosing acute PJI, especially hematogenous ones, but proves less effective in identifying low-grade PJI, which remain a diagnostic challenge [38].

Another M-PCR panels that have been evaluated for the diagnosis of PJI include Unyvero i60 [39,40]. Unlike the BioFire panel, this assay includes *S. epidermidis* but not *C. acnes*, limiting its ability to detect infections caused by the latter.

Recently, digital droplets PCR (ddPCR) has sparked considerable interest in the clinical field [41], particularly in infectious disease research [42]. When compared to the real-time PCR, in fact, this approach demonstrated superior sensitivity, exhibiting a limit of detection approximately tenfold lower. As such, ddPCR-based assays constitutes a highly promising diagnostic modality with significant utility in the identification of bacterial pathogens in patients with PJI [43]. To date, however, no studies have clearly defined the performance of ddPCR in the diagnosis of low-grade PJI caused by *C. acnes* or other fastidious organisms.

#### 2.2.2. Next-Generation Sequencing (NGS)

Next-Generation Sequencing (NGS) encompasses high-throughput technologies that allow rapid and comprehensive genetic analysis, increasingly applied in infectious disease diagnostics. Two main approaches are currently employed in clinical microbiology: targeted NGS (tNGS) and metagenomic NGS (mNGS) (Figure 2 and Table 1) [44]. tNGS focuses on specific genomic regions, such as bacterial 16S rRNA, providing high sensitivity for known pathogens with reduced cost and faster turnaround time [45]. However, this targeted approach does not provide information on resistance genes or virulence factors, which limits its value for therapeutic guidance. By contrast, mNGS sequences all nucleic acids within a sample without prior target selection, offering hypothesis-free identification of bacteria, fungi, viruses, and parasites [46]. This approach is particularly advantageous in culture-negative or polymicrobial infections and has demonstrated superior performance to conventional culture in detecting fastidious organisms such as *Cutibacterium acnes* in PJI [47]. Moreover, mNGS has the potential to identify antimicrobial resistance mechanisms and virulence determinants. Nonetheless, several challenges remain, including the overwhelming proportion of host DNA that can obscure pathogen signals, the risk of contamination, and the need for curated reference databases and specialized bioinformatics expertise for data interpretation [48]. Despite these limitations, accumulating evidence supports the clinical utility of NGS in PJI. A recent meta-analysis indicated that mNGS achieved the highest diagnostic odds ratio among molecular methods; however, heterogeneity in study design, technique execution, and specimen selection limits the generalizability of these findings [49]. In this regard, comparative studies suggest that sonication fluid may represent the most informative specimen type for mNGS in PJI, outperforming both synovial fluid and peri-prosthetic tissue in terms of pathogen recovery [50].

Despite these advances, the precise role of NGS within the diagnostic algorithm for PJI has not yet been fully established. A potential role has recently been proposed for its use as a second-line diagnostic tool. Current recommendations suggest that NGS should be considered in acute PJI cases when both culture and M-PCR results are negative. Similarly, in chronic infections, NGS is not recommended as a first-line approach but should instead be reserved for culture-negative cases, given the increased risk of false-positive findings associated with its high sensitivity [62].

### 2.3. Biomarkers

Biomarkers play a pivotal role in enhancing the diagnostic accuracy of PJI, particularly in cases where conventional microbiological methods yield inconclusive results [63]. In this context, biomarkers offer an adjunctive tool by reflecting the underlying biological processes—either host immune responses or microbial metabolism—occurring at the site of infection. Biomarkers used in the diagnosis of PJI can be broadly classified into three categories: pathogen-derived and host-derived biomarkers, the latter further subdivided into serum and synovial biomarkers.

#### 2.3.1. Pathogen-Derived Biomarkers

Among biomarkers derived or produced by micro-organisms, D-lactate has garnered attention as a promising analyte. As a D-(−)-lactic acid stereoisomer produced almost exclusively by bacterial metabolism during fermentation, D-lactate is not synthesized in significant amounts by human cells and, consequently, it is a more selective indicator of bacterial presence.

A recent in vitro study by Morovic et al., confirmed that a wide range of clinically relevant bacteria produce substantial amounts of D-lactate, whereas human tissues and fluids contain negligible levels, reinforcing its specificity as an infection marker [64].

This foundational work has been supported by clinical evidence: in a prospective observational study, Yermak et al. evaluated synovial D-lactate levels in patients undergoing revision arthroplasty and reported a sensitivity of 90% and specificity of 83% for the diagnosis of PJI, a diagnostic performance comparable to that of synovial white blood cell count, one of the established gold-standard tests in this setting [65].

Further advancing its clinical utility, Karbysheva et al. demonstrated that D-lactate levels vary significantly depending on the bacterial species, suggesting that this biomarker may not only aid in diagnosing PJI, but also provide indirect information on the underlying pathogen, with potential implications for guiding early empirical therapy [66].

In line with these findings, Fuchs et al. identified an optimal diagnostic cut-off of 0.04 mmol/L in synovial fluid, which yielded a sensitivity of 90.7% and specificity of 83.3%, thereby underscoring the potential of D-lactate as a rapid, low-cost, and accurate diagnostic adjunct in the PJI setting [67].

The growing interest in D-lactate has prompted meta-analytical evaluation. In a systematic review and meta-analysis, Li et al. synthesized data from multiple studies and reported a pooled sensitivity of 82% and specificity of 76%, with an area under the ROC curve (AUC) of 0.84, confirming its moderate-to-high diagnostic accuracy and supporting its potential as a useful adjunct in PJI workup [68].

Nevertheless, the lack of standardized cut-off values, which may vary depending on the pathogen’s virulence [64], highlight the need for further validation before this test can be widely implemented in clinical practice.

#### 2.3.2. Host’s Biomarkers

Host-derived biomarkers play a crucial role in the diagnostic workup of PJI, particularly in differentiating infectious from aseptic failure. In response to infection, the host mounts a complex local and systemic immune reaction, characterized by the release of a variety of inflammatory mediators, acute-phase proteins, and cellular effectors. These immunological changes can be quantitatively or qualitatively assessed through specific biomarkers detectable in serum or synovial fluid.

##### Serum and Plasma Biomarkers

Due to their accessibility, serological markers serve as a key component in the initial assessment of patients with suspected PJI [69]. Accordingly, they are often employed as the first-line investigation in individuals presenting with painful joint prostheses. Nonetheless, these markers may be elevated in a variety of non-infectious conditions, particularly in patients with underlying medical comorbidities, which can limit their specificity [70].

The serum white blood cell (WBC) count is a routinely used marker of systemic inflammation, but its diagnostic utility in PJI is limited. Studies have shown low sensitivity and moderate specificity, with minimal correlation to synovial fluid WBC levels [71,72]. Moreover, evidence from large prospective cohorts indicates that serum WBC count is not an independent predictor of PJI, further underscoring its restricted utility [73].

The neutrophil-to-lymphocyte ratio (NLR) has been investigated as a simple and accessible inflammatory marker for PJI. A meta-analysis including 12 studies reported a pooled sensitivity of 73% and specificity of 75%, with an AUC of 0.79, supporting its moderate diagnostic value [74]. In chronic PJI, an NLR cut-off around 2.56 showed 57% sensitivity and 78% specificity, although its performance was not improved when combined with other inflammatory markers [75]. In contrast, studies on early post-operative infections reported better accuracy, with cut-offs near 2.77 yielding 85% sensitivity and 90% specificity [76].

However, current research on the diagnostic role of NLR in PJI remains preliminary, and further large-scale studies are required to better establish its clinical relevance.

C-reactive protein (CRP) and erythrocyte sedimentation rate (ESR) are routinely employed as initial screening tools due to their broad accessibility and cost-effectiveness [77]. Nonetheless, their application is limited by relatively low specificity—given that levels may rise in other inflammatory or post-operative states—and by the absence of well-defined diagnostic thresholds for PJI [78]. Furthermore, PJI caused by low-virulence organisms may not elicit a significant systemic inflammatory response, and as a result, ESR and CRP levels can remain within normal ranges. Therefore, normal values of these markers should not be considered sufficient to exclude the presence of PJI [79].

Recent evidence suggests that serum fibrinogen is another promising serum biomarker. In a retrospective study of 156 patients undergoing revision arthroplasty, a fibrinogen threshold of 4.20 g/L yielded a sensitivity of 86%, specificity of 90%, and an AUC of 0.916, outperforming CRP, ESR, and serum WBC count [80]. In a single-center series involving total knee arthroplasty revisions, a fibrinogen threshold of 420 mg/dL (4.20 g/L) achieved 67% sensitivity and 82% specificity in distinguishing PJI from aseptic failure, and specificity increased to 90% when combined with a D-dimer level > 1063 ng/mL [81]. Another study demonstrated serum fibrinogen’s sensitivity of 79.3% and specificity of 94.6% (AUC 0.928), outperforming CRP and ESR in differentiating PJI and aseptic loosening [82]. Although fibrinogen shows promising performance, pooled data indicate it should serve not as a standalone test but as an adjunct to CRP, ESR, and synovial fluid analysis [63]. Its high specificity makes it especially useful for ruling out infection, while sensitivity varies across cohorts and clinical settings.

Although serum procalcitonin (PCT) has been studied as a biomarker for PJI, meta-analytical evidence indicates it has limited clinical utility, exhibiting low sensitivity (~0.44) despite reasonable specificity (~0.85), and thus is not recommended as a single diagnostic indicator [83].

Serum cytokines, particularly IL-6, IL-1β, and TNF-α, serve as important adjunctive biomarkers. Among these, IL-6 exhibits notable diagnostic value: meta-analyses report pooled sensitivity ~0.72 and specificity ~0.88 (AUC ~0.88) [84]. In acute PJI, a serum IL-6 threshold near 7.2 pg/mL achieves sensitivity of 93.5% and specificity of 83.6% [85].

IL-1β and TNF-α levels are also elevated in septic cases; however, their diagnostic performance remains less well characterized compared to IL-6 [86]. Early serum-based studies indicate that IL-6 concentrations above approximately 12 pg/mL achieve high sensitivity (about 95%) and specificity (around 87%) for distinguishing septic from aseptic prosthetic failures, and concomitant elevation of TNF-α may further support suspicion of infection [87].

The use NGS platforms is rapidly gaining ground in the field of biomarker discovery, including the profiling of serum and plasma microRNAs, small non-coding RNA molecules (~18–24 nucleotides) that regulate post-transcriptional gene expression and are stable in serum and plasma. Because of their involvement in inflammatory and immune responses, miRNAs are increasingly being investigated as novel biomarkers in infectious and inflammatory diseases, including PJI.

In a prospective pilot study by Paksoy et al., several miRNAs—including hsa-miR-21-3p, hsa-miR-1290, hsa-miR-4488, and hsa-miR-1260a—were found to be significantly upregulated in patients with high-grade hip PJI. Notably, their expression levels showed a strong positive correlation with serum CRP concentrations, suggesting that they reflect systemic inflammatory activity and may serve as complementary markers to traditional acute-phase reactants [88].

NGS allows high-throughput, unbiased detection of low-abundance miRNA species and offers a powerful tool for identifying complex biomarker signatures with diagnostic and prognostic relevance. As these technologies become more accessible and clinically validated, they are likely to play a pivotal role in expanding the biomarker landscape for PJI and other orthopedic infections.

##### Synovial Biomarkers

Synovial biomarkers have emerged as highly valuable tools in the diagnosis of PJI, offering superior diagnostic accuracy compared to traditional serum markers. Unlike systemic biomarkers, synovial markers provide a more localized and specific reflection of the intra-articular inflammatory response. This is particularly relevant in PJI, where changes in the synovial microenvironment occur earlier and more directly than in the systemic circulation [89].

Synovial fluid leukocyte analysis—specifically total WBC and polymorphonuclear neutrophil percentage (PMN %)—represents a highly accurate diagnostic approach. In chronic PJI, a comprehensive meta-analysis demonstrated a diagnostic odds ratio (DOR) of 58.4 for WBC (AUC = 0.952) and 43.2 for PMN % (AUC = 0.941), with optimal thresholds around 2600 cells/µL for WBC and 70% for PMN %—offering excellent sensitivity and specificity; rule-in thresholds (specificity > 95%) were WBC ≥ 3000 cells/µL and PMN % ≥ 75%, while rule-out thresholds (sensitivity > 95%) were ≤1500 cells/µL and ≤65% [90,91].

Gramlich et al. confirmed these findings in a prospective cohort (*n* = 405), identifying optimal cut-offs of 2479 cells/µL and 67% PMN, noting that existing ICM/EBJIS criteria favor high specificity at the expense of sensitivity [92].

In the context of acute post-operative PJI, synovial WBC outperformed PMN %, with DORs of 123.6 (AUC = 0.96) vs. 18.7 (AUC = 0.88) [93].

Across acute and chronic contexts, synovial WBC and differential counts remain highly reliable, yet accuracy can be influenced by confounders such as inflammatory arthropathies or metal debris, reinforcing the need for standardized thresholds and clinical integration with other diagnostic modalities [94].

Recent advancements in multi-omics approaches have enabled the comprehensive characterization of the joint microenvironment, thereby identifying key biomarkers implicated in septic failure. Notable, among these: C reactive protein (CRP), interleukin-6 (IL-6), alpha-defensin, calprotectin, leukocyte esterase (LE), and myeloperoxidase (MPO) (Table 2) [89].

Recent studies have demonstrated that synovial CRP offers robust diagnostic accuracy for PJI [95,96]. A multicentre cohort analysis (*n* ≈ 588), published in 2022, reported a sensitivity of 74.2% and specificity of 98.0% (AUC 0.951; 95% CI 0.932–0.970) for synovial CRP—in contrast to serum CRP’s lower specificity (~88%)—highlighting superior performance in confirming PJI [97]. A single-centre validation of over 4000 synovial fluid samples identified an optimal diagnostic threshold of 4.45 mg/L, yielding sensitivity 86.1% (95% CI 85.0–87.1%) and specificity 87.1% (95% CI 86.7–87.5%) (AUC 0.929) [98]. Another prospective cohort involving 97 revision cases determined a cut-off of 7.26 mg/L for chronic PJI, achieving 84.6% sensitivity and 93.1% specificity (AUC 0.937) [99]. When assessed according to the updated EBJIS diagnostic criteria, synovial CRP demonstrated an optimal diagnostic threshold of 2.7 mg/L. Notably, concentrations exceeding 8 mg/L were strongly associated with high specificity and a high positive predictive value, reinforcing the biomarker’s utility in confirming PJI [100]. Collectively, recent evidence, supports synovial CRP as a reliable adjunctive biomarker, with diagnostic metrics rivaling serum CRP and outperforming it in specificity—especially when incorporated into combined diagnostic strategies [99,101].

Synovial IL-6 has emerged as a highly accurate biomarker for the diagnosis of PJI in hip and knee arthroplasty. A meta-analysis of 30 diagnostic accuracy studies reported a pooled sensitivity of 87% and specificity of 90% (AUC 0.94; 95% CI 0.92–0.96) for synovial IL-6, outperforming serum IL-6 (sensitivity 76%, specificity 88%, AUC 0.88) [84]. A prospective cohort study by Qin et al. established a synovial IL-6 cut-off of ~1855 pg/mL, achieving sensitivity of 94.6% and specificity of 92.9%, and when combined with serum IL-6, reached a diagnostic accuracy of 96.8% [102]. Similarly, Li et al. confirmed in their pooled analysis that synovial IL-6 demonstrates superior diagnostic odds ratio (DOR = 57) over serum IL-6 (DOR = 22), with likelihood ratios indicating strong rule-in and rule-out utility [84]. While the invasive nature and variable cut-off thresholds have been noted as limitations, clinical reviews emphasize that synovial IL-6 maintains greater diagnostic performance than conventional markers (e.g., CRP, ESR) and warrants integration into diagnostic algorithms for PJI [63].

Synovial α-defensin has emerged as one of the most accurate biomarkers for the diagnosis of PJI. Among all currently available synovial biomarkers, α-defensin is the only one formally incorporated into both American and European diagnostic algorithms [9,103]. Laboratory-based α-defensin immunoassays (ELISA) demonstrate pooled sensitivity and specificity exceeding 96%, with diagnostic odds ratios surpassing 800 and area under the ROC curve (AUC) close to 0.99, representing the highest diagnostic performance among all synovial markers [104]. Although lateral flow versions (e.g., Synovasure) offer rapid intraoperative results, they are slightly less sensitive while maintaining high specificity [105,106]. Notably, α-defensin levels are not significantly influenced by prior antibiotic therapy or making the test reliable even in diagnostically complex scenarios [107]. However, its use is most appropriate as a rule-in tool in cases with intermediate or high clinical suspicion, rather than as a primary screening method [108]. The risk of false positives in cases of metallosis (an adverse tissue reaction caused by accumulation of metal debris originating from friction of joint prosthesis components or osteosynthetic material), inflammatory systemic conditions [109], recent trauma and surgery [110] and false negatives in chronic low-grade infections with draining sinuses [105], necessitates integration with other synovial biomarkers for optimal diagnostic accuracy [110]. Taken together, current high-quality evidence confirms that synovial α-defensin—especially when measured via ELISA—should be viewed as a second-tier confirmatory test, currently unmatched in its diagnostic utility for PJI.

LE represents another useful biomarker in the diagnostic evaluation of PJI. Neutrophils recruited to the infected joint, release LE into the synovial fluid, which can be detected through colorimetric assays based on enzyme-induced color changes [111]. The test is typically performed using reagent strips originally designed for urine analysis, requiring only a small sample of synovial fluid. Multiple studies have reported that LE testing offers high specificity, along with strong positive and negative predictive values, although its sensitivity is generally considered moderate [112]. In a study by Wetters et al., LE strip testing demonstrated a sensitivity ranging from 92% to 100% and a specificity close to 90%. Nevertheless, the reliability of the test can be compromised by the presence of blood or debris in the synovial fluid, as these contaminants may interfere with the colorimetric reaction and thus hinder accurate interpretation. However, a critical limitation of the LE strip test is its susceptibility to interference from hemoglobin and other cellular components in bloody or contaminated synovial fluid. Since the assay relies on a visual colorimetric reaction, the presence of blood can obscure or alter the color change, leading to inconclusive or false results. Therefore, LE testing is recommended only for non-hematic, clear synovial fluid samples to ensure accurate interpretation and avoid diagnostic ambiguity [113].

Synovial calprotectin has shown high diagnostic accuracy in detecting PJI, with several studies supporting its potential as a reliable biomarker. For instance, a prospective study by Grassi et al. demonstrated that calprotectin measured via immunoassay in synovial fluid achieved a sensitivity of 100% and specificity of 95%, with an AUC of 0.996, indicating near-perfect discriminative power [114]. Similarly, Wouthuyzen-Bakker et al. found that using a lateral flow assay with a 50 mg/L cutoff yielded a sensitivity of 89%, specificity of 90%, and a particularly high negative predictive value (NPV) of 97% in chronic infections [115]. More recently, a large prospective multicenter study by Ruffier d’Epenoux et al. further confirmed the diagnostic utility of synovial calprotectin—together with synovial CRP—for chronic PJI, reinforcing its role as a robust adjunctive biomarker in clinical practice [116].

Meta-analyses reinforce these findings: one pooling data from seven studies reported pooled sensitivity and specificity of 94% and 93%, respectively, with a diagnostic odds ratio (DOR) of 222.32 and an AUC of 0.98 [117]. A more recent and comprehensive meta-analysis including 14 studies and 902 patients showed similar pooled sensitivity (92%) and specificity (93%), with ELISA outperforming lateral flow in terms of DOR (907 vs. 114) and AUC (0.968 vs. 0.915) [118].

The major strengths of calprotectin include its rapid turnaround time (especially with lateral flow devices), low cost, and minimal sample volume requirement, making it particularly valuable in intraoperative or point-of-care settings. Its performance remains robust even in the presence of mild systemic inflammation, and it shows minimal cross-reactivity with metal wear debris or hemarthrosis, unlike some traditional biomarkers [119].

However, limitations must be acknowledged. Its diagnostic accuracy may be reduced in early postoperative settings or in patients with inflammatory arthropathies (e.g., rheumatoid arthritis), where elevated synovial calprotectin levels can yield false positives [120]. Moreover, while ELISA offers superior accuracy, it requires laboratory infrastructure and longer processing times compared to lateral flow methods. Additionally, a universally accepted cut-off has not been established, leading to heterogeneity across studies and hindering standardization in clinical practice [118].

Synovial fluid MPO is emerging as a useful biomarker in differentiating both acute and low-grade PJI from aseptic failures. A previous study by our group evaluated active MPO levels in 99 synovial fluid samples—65 PJI (33 acute, 32 low-grade) versus 34 aseptic failures—and found significantly elevated MPO in PJI (*p* < 0.0001). ROC analysis showed an AUC of 0.86 (95% CI: 0.78–0.93); at a threshold of 561.9 U/mL, active MPO reported sensitivity of 69%, specificity of 88%, accuracy of 75.8%, PLR of 5.88, and NLR of 0.35 [121]. Earlier, an analogous study by Kimura and colleagues, with a smaller cohort (19 chronic PJI vs. 18 aseptic failures) similarly demonstrated significantly higher MPO levels in infected joints, supporting its discriminative potential despite sample size limitations [122].

Advantages of synovial MPO include its reflection of neutrophil-mediated host response, making it particularly helpful in detecting culture-negative or low-grade infections. The assay is relatively affordable and practical for specialized orthopedic diagnostics [121]. However, its diagnostic sensitivity (~69%) remains moderate, which raises concerns about the potential for false-negative results. In addition, most existing studies are limited by small sample sizes and single- or dual-center designs, which constrain the generalizability of their findings. The proposed cut-off values also lack external validation, further limiting clinical applicability. While preliminary data suggest comparable performance in both acute and chronic PJI, this observation requires confirmation through stratified analyses. To support broader clinical adoption, larger multicentre investigations and direct comparisons with established biomarkers—such as α-defensin and calprotectin—are warranted.

As summarized in Table 3, a concise overview of currently available synovial biomarkers for the diagnosis of PJI is provided, highlighting their applicability as rapid intra-operative tests and relative costs, thereby facilitating a critical appraisal of their clinical utility and supporting evidence-based decision-making in the perioperative setting.

To date, no single biomarker has demonstrated sufficient diagnostic accuracy to serve as a standalone test for the diagnosis of PJI. Although individual biomarkers have shown promising sensitivity and specificity, their diagnostic reliability is limited when evaluated independently [123]. Recent evidence suggests that simultaneous quantification of multiple synovial biomarkers may significantly enhance diagnostic performance. Technological advances have enabled the development of rapid multiplex micro-ELISA platforms enabling the simultaneous detection of multiple synovial biomarkers with minimal sample volumes and short turnaround times. A recently validated micro-ELISA system demonstrated promising accuracy for the simultaneous measurement of inflammatory mediators and acute-phase proteins in synovial fluid, delivering results within 30 min and showing potential clinical utility in the context of PJI diagnosis [124].

Similarly, a novel multiplex and highly sensitive lateral flow immunoassays platform has been developed, offering portable, low-cost, and clinically applicable diagnostic potential for PJI [125].

### 2.4. Artificial Intelligence

Artificial intelligence (AI) has rapidly emerged as a promising adjunct in the field of periprosthetic joint infection (PJI), spanning applications from automated case detection to diagnostic classification and outcome prediction. Natural language processing has demonstrated utility in extracting relevant data elements from clinical records, facilitating surveillance and research [126]. Predictive models integrating perioperative variables, laboratory findings, and synovial fluid biomarkers have shown strong performance in identifying patients at high risk of failure following irrigation and debridement [127,128,129,130]. Similarly, machine learning frameworks leveraging national databases or registry data provide scalable approaches to risk stratification, though external validation remains essential [131,132].

Recent advances extend to imaging and pathology: AI applied to dynamic bone scintigraphy and radiographs can support differentiation between infection and aseptic failure [133,134], while deep-learning models trained on digitized histology slides achieve excellent diagnostic accuracy [135]. Systematic reviews highlight the breadth of these innovations and suggest a growing role for AI not only in diagnosis but also in prevention and clinical decision support [136,137]. However, issues of interpretability, dataset heterogeneity, and bias require careful attention. Furthermore, GPT-based chatbots currently lack reliability for clinical decision-making and should be confined to educational or supportive roles [138,139,140].

Overall, while AI offers the potential to complement culture-based and biomarker-driven diagnostics, robust prospective validation and integration into clinical workflows are necessary before these tools can be routinely adopted. If successfully implemented, AI may transform the management of PJI by enabling earlier diagnosis, personalized risk stratification, and more precise treatment strategies.

## 3. Conclusions

The diagnostic landscape of PJI has rapidly expanded beyond conventional culture-based methods, incorporating molecular, immunological, and computational innovations. Techniques such as PCR and NGS have improved the detection of fastidious or biofilm-associated pathogens, particularly in culture-negative cases, while synovial biomarker profiling continues to refine diagnostic accuracy through multiplex platforms. AI and machine learning models are emerging as integrative frameworks capable of synthesizing clinical, microbiological, and biomarker data to support predictive diagnostics and individualized therapeutic decision-making.

Looking ahead, several challenges and opportunities should guide future research. First, the standardization and clinical validation of molecular and NGS assays across laboratories remain essential for their broader adoption. Second, prospective, multicenter trials are needed to define robust biomarker panels, ideally tailored to diverse patient populations and implant types. Third, the development of interpretable, regulation-compliant AI systems that can integrate multimodal data and provide real-time decision support will be critical to ensure clinical trust and adoption. Finally, efforts should extend toward cost-effectiveness analyses and implementation studies, which will determine how these advanced diagnostics can be incorporated into routine clinical pathways without overburdening healthcare systems.

By addressing these forward-looking challenges, the convergence of molecular diagnostics, synovial biomarker analytics, and AI-driven platforms has the potential to not only improve diagnostic precision but also to reshape the clinical management of PJI, enabling earlier interventions, tailored treatment strategies, and ultimately better long-term outcomes for patients.

## Figures and Tables

**Table 1:** Original research articles evaluating the use of NGS in the diagnosis of PJI.

Authors	Nr. of PJIs Analyzed	PJI Diagnostic Criteria	Type of Sample	Type of NGS	Sensitivity (%)	Specificity (%)	Reference
Huang et al.	49	MSIS	Synovial fluid	Metagenomic (shotgun)	95.9	95.2	[51]
Kildow et al.	116	MSIS	Synovial fluid; Peri-prosthetic tissue swabs	Targeted 16S rRNA	60.9	89.9	[52]
Flurin et al.	47	IDSA	Sonicate fluid	Targeted 16S rRNA	85.0	98.0	[53]
Fang et al.	25	MSIS	Synovial fluid	Metagenomic (shotgun)	92.0	91.7	[54]
Cai et al.	22	MSIS	Periprosthetic tissue	Metagenomic (shotgun)	95.45	90.91	[55]
Hao et al.	58	MSIS	Synovial fluid; Periprosthetic tissue	Metagenomic (shotgun)	94.8	89.2	[56]
Shi et al.	46	ICM 2018	Synovial fluid; Periprosthetic tissue	Metagenomic (shotgun)	89.13	94.74	[57]
Yin et al.	15	MSIS	Synovial fluid	Metagenomic (shotgun)	93.3	90.0	[58]
Tan et al.	43	MSIS	Synovial fluid; Periprosthetic tissue; Sonicate fluid	Metagenomic (shotgun) on synovium, tissues, sonicate	93.0	94.4	[50]
Tarabichi et al.	28	MSIS	Periprosthetic tissues	Targeted (multi-locus amplicon)	89.3	73.0	[22]
Azad et al.	60	IDSA	Synovial fluid	Targeted 16S rRNA	96.0	94.0	[59]
Wang et al.	63	MSIS	Joint fluid; Sonication fluid; Homogenized tissue	Metagenomic (shotgun)	95.6	94.4	[60]
Flurin et al.	154	IDSA	Synovial fluid	16S rRNA gene-based PCR followed by Sanger sequencing and/or tMGS	83	100	[61]

Note: MSIS, Musculoskeletal Infection Society; IDSA, Infectious Diseases Society of America; ICM 2018, The 2018 International Consensus Meeting; NGS, next-generation sequencing.

**Table 2:** Host synovial biomarkers associated with periprosthetic joint infection, categorized by biological function.

Category	Biomarkers
Acute phase proteins	C-reactive protein (CRP), Haptoglobin, Complement (C1q, C3b/C3i, C4b, C5, C5a, MBL, properdin), D-dimer, Lactotransferrin
Cytokines and chemokines	Interleukin-6 (IL-6), Interleukin-1 alpha (IL-1α), Interleukin-1 beta (IL-1β), Interleukin-5 (IL-5), Interleukin-8 (IL-8), Interleukin-10 (IL-10), Interleukin-17A (IL-17A), Interleukin-18 receptor 1 (IL-18R1), C-C motif chemokine ligand 2 (CCL2), CCL3, CCL4, CCL20, C-X-C motif chemokine ligand 1 (CXCL1), CXCL2, CXCL5, CXCL6, Tumor necrosis factor (TNF), Interferon gamma (IFN-γ)
Enzymes and proteases	Alpha-defensin, Myeloperoxidase (MPO), Cathepsin G, Elastase-2 (ELA-2), Matrix metalloproteinase-1 (MMP-1), Matrix metalloproteinase-9 (MMP-9), Lysozyme C, Presenilin, Oncostatin M, Osteopontin, Procalcitonin, Presepsin, Thrombospondin
Cellular/inflammatory markers	Calprotectin, Leukocyte esterase (LE), Bactericidal permeability-increasing protein (BPI), EN-RAGE, ERN1, Coagulation factor VII (F7), FCRL4, Granulocyte colony-stimulating factor (G-CSF), CD40 ligand (CD40L), Lamin-B1, Leucine-rich alpha-2-glycoprotein (LRG1), Lipocalin, Pyruvate glycogen phosphorylase (PYGL), Ribonuclease 3 (RNASE3), Toll-like receptor 2 (TLR-2)

**Table 3:** Biochemical and immunological synovial biomarkers for the diagnosis of PJI: availability of intra-operative point-of-care tests and relative costs.

Biomarker	Rapid Intraoperative Test (POCT)	Relative Cost per Test	Notes
C-reactive protein (CRP)	No validated intraoperative POCT available; measurement requires laboratory-based assays (immunoturbidimetry/ELISA).	Low	Synovial CRP has diagnostic value but is less specific than α-defensin or calprotectin.
Interleukin-6 (IL-6)	No rapid POCT; determination is usually performed by ELISA or automated immunoassays.	Moderate–High	Reported as a sensitive marker of synovial inflammation with good diagnostic performance in meta-analyses.
Leukocyte esterase (LE)	Yes—urine dipstick–based strip test, readable intraoperatively within minutes.	Low	Widely studied as a low-cost, rapid screening tool; useful particularly to rule out infection.
α-defensin	Yes—lateral flow assay (e.g., Synovasure) provides results within 10–20 min; ELISA format also available (laboratory-based).	High	Among the most validated synovial biomarkers, with high specificity for PJI.
Calprotectin	Yes—lateral flow POCT (e.g., Lyfstone), result within ~15 min.	Moderate	Increasing evidence supports its accuracy; CE-marked for POC use.
Myeloperoxidase (MPO)	Not yet standard—prototype rapid assays under evaluation; not widely commercially available.	Low–Moderate	Emerging biomarker with promising diagnostic performance but limited clinical validation to date.

Note. POCT = point-of-care test. Cost categories are relative and indicative: Low (<€5 per test), Moderate (€5–€50), High (>€50–€100). Costs may vary according to country, supplier, and institutional agreements. Intraoperative POCT refers to assays providing results within minutes and suitable for decision-making during surgery.

## Data Availability

Not applicable.

## Abbreviations

The following abbreviations are used in this manuscript:

PJI	Periprosthetic joint infection
PCR	Polymerase chain reaction
NGS	Next-generation sequencing
AI	Artificial intelligence
EBJIS	European Bone and Joint Infection Society
IMC	Isothermal microcalorimetry
ML	Machine learning
CNNs	Convolutional neural networks
SVM	Support Vector Machine
XGBoost	Extreme Gradient Boosting
DNA	Deoxyribonucleic Acid
BR-PCR	Broad-range PCR
qPCR	Quantitative PCR
M-PCR	Multiplex PCR
ddPCR	Digital droplets PCR
tNGS	Targeted next-generation sequencing
mNGS	Metagenomic next-generation sequencing
rRNA	Ribosomal ribonucleic acid
MSIS	Musculoskeletal Infection Society
IDSA	Infectious Diseases Society of America
ICM	International Consensus Meeting
ROC	Receiver Operating Characteristic curve
AUC	Area Under the Curve
WBC	White blood cell
NLR	Neutrophil-to-lymphocyte ratio
CRP	C-reactive protein
ESR	Erythrocyte sedimentation rate
PCT	Procalcitonin
IL	Interleukin
IL-18R1	Interleukin-18 receptor 1
MMP-1	Matrix metalloproteinase-1
CCL	C-C motif chemokine ligand
CXCL	C-X-C motif chemokine ligand
TNF-α	Tumor Necrosis Factor Alpha
BPI	Bactericidal permeability-increasing protein
F7	Coagulation factor VII
G-CSF	Granulocyte colony-stimulating factor
LRG1	Leucine-rich alpha-2-glycoprotein
PYGL	Pyruvate glycogen phosphorylase
RNASE3	Ribonuclease 3
TLR-2	Toll-like receptor 2
PMN %	Polymorphonuclear neutrophil percentage
DOR	Diagnostic odds ratio
LE	Leukocyte esterase
MPO	Myeloperoxidase
ELISA	Enzyme-linked immunosorbent assay
NPV	Negative predictive value
PLR	Positive likelihood ratio
NLR	Negative likelihood ratio
LLM	Large language model
POCT	Point-of-care test
